# A Method for Human Facial Image Annotation on Low Power Consumption Autonomous Devices

**DOI:** 10.3390/s20072140

**Published:** 2020-04-10

**Authors:** Tomasz Hachaj

**Affiliations:** Institute of Computer Science, Pedagogical University of Krakow, 2 Podchorazych Ave, 30-084 Krakow, Poland; tomekhachaj@o2.pl

**Keywords:** facial image annotation, RGB camera sensor, neural network, deep neural network, eigenfaces, nearest neighbour classifier, low power consumption computer

## Abstract

This paper proposes a classifier designed for human facial feature annotation, which is capable of running on relatively cheap, low power consumption autonomous microcomputer systems. An autonomous system is one that depends only on locally available hardware and software—for example, it does not use remote services available through the Internet. The proposed solution, which consists of a Histogram of Oriented Gradients (HOG) face detector and a set of neural networks, has comparable average accuracy and average true positive and true negative ratio to state-of-the-art deep neural network (DNN) architectures. However, contrary to DNNs, it is possible to easily implement the proposed method in a microcomputer with very limited RAM memory and without the use of additional coprocessors. The proposed method was trained and evaluated on a large 200,000 image face data set and compared with results obtained by other researchers. Further evaluation proves that it is possible to perform facial image attribute classification using the proposed algorithm on incoming video data captured by an RGB camera sensor of the microcomputer. The obtained results can be easily reproduced, as both the data set and source code can be downloaded. Developing and evaluating the proposed facial image annotation algorithm and its implementation, which is easily portable between various hardware and operating systems (virtually the same code works both on high-end PCs and microcomputers using the Windows and Linux platforms) and which is dedicated for low power consumption devices without coprocessors, is the main and novel contribution of this research.

## 1. Introduction

Facial image analysis and classification is among the most important and up-to-date tasks in computer vision, signal processing, and pattern recognition methods. By looking at a human face, we can identify not only the most general features, such as gender, race, and age, but also facial expression (emotions) and recollect their identity (i.e., whether that person was met before). The ability of facial image analysis is basic and is among the most important human-to-human interaction processes. Lately, due to the development of novel machine learning algorithms (especially deep learning) and access to large data sets of facial images, it has become possible to automate some facial recognition approaches by using computer methods. In the following subsections, various applications of those algorithms and the contributions of this paper will be discussed.

### 1.1. Face Recognition

Before facial images can be processed, there are several pre-processing steps that have to be done. Traditionally, one needs to cascade two blocks: face localization and facial descriptor construction [1]. In some studies, these steps are replaced by applying a single deep neural network (DNN) architecture [2,3]. Facial recognition systems were applied in many computer systems, from real-time face tracking and recognition systems [4] and identification of people in personal smartphone galleries [5] to large-scale classification and security systems [6]. Chang et al. [7] proposed a facial recognition algorithm based on a support vector machine (SVM) combined with Visual Geometry Group (VGG) network model for extracting facial features, which not only accurately extracts face features, but also reduces feature dimensions and avoids irrelevant features in the calculation. However, some researchers continue to use the well-known eigenfaces approaches, using the Euclidean distance measure [8]. Among the up-to-date problems in face recognition is the age-invariant face recognition task [9]. At the same time, the tactics used to mislead these systems have become more complex and counter-measure approaches are necessary. For example, Lucena et al. [10] proposed an approach based on transfer learning with a pre-trained convolutional neural network (CNN) model, which uses only static features to recognize photo, video, or mask attacks.

### 1.2. Face Attribute Prediction and Annotation

A facial image can be described (annotated) with many important features, such as gender, race, shape of the chin, hair colour, and so on. Zhong et al. [1], Aly et al. [11], and Fan et al. [12] achieved these goals using descriptors constructed from different levels of the CNNs for different attributes to best facilitate face attribute prediction. Yilmaztürk et al. [13] studied automatic and online face annotation for personal videos/episodes of TV series considering Nearest Neighbour, Linear discriminant analysis (LDA), and SVM classification with Local Binary Patterns, Discrete Cosine Transform, and Histogram of Oriented Gradients feature extraction methods, considering their recognition accuracies and execution times.

Facial image annotation can be also applied in content-based image retrieval (CBIR). These types of systems find images within a database that are similar to query. Conilione et al. [14], Nguyen et al. [15], and Wang et al. [16] attempted to discover semantic labels for facial features for use in such systems.

Chang et al. [17] described the use of an unsupervised label refinement (ULR) method for the task of fixing weakly labelled facial image data collected from the Internet with mislabelled images. To improve the correction accuracy of ULR, particle swarm optimization (PSO) and binary particle swarm optimization (BPSO) were used to solve the binary constraint optimization task in that study. Firmino et al. [18] used automatic and semi-automatic annotation techniques for people in photos using the shared event concept, which consists of many photos captured by different devices of people who attended the same event. Among the most popular and efficient approaches for facial image annotation are various convolutional neural networks (CNNs) [1,19,20,21,22].

Additional large surveys of describable visual face attributes which can be applied in face recognition and annotation can be found in [23,24].

### 1.3. Other Computer Methods That Use Facial Images

Among the other methods based on facial images, we mention face completion methods, the goal of which is to reconstruct facial image from incomplete or obscure data. Zhang et al. [25] reported using a symmetry-aware face completion method based on facial structural features found using a deep generative model. The model is trained with a combination of a reconstruction loss, a structure loss, two adversarial losses, and a symmetry loss, which ensures pixel faithfulness, local–global content integrity, and symmetric consistency. Low-resolution facial images might be also used as input for attribute-guided face generation systems [26]. The massive potential of face recognition methods poses a serious privacy threat to individuals who do not want to be profiled. Chhabra et al. [27] and Flouty et al. [28] presented novel algorithms for anonymizing selective attributes which an individual does not want to share, without affecting the visual quality of facial images.

### 1.4. Contributions of This Research

Due to ongoing miniaturization of the computer hardware, there are many affordable low power consumption microcomputer platforms which can be used for general purpose computing. Low power consumption means hardware-powered; for example, by USB standard (5 V, 3 A). Such platforms typically have limited computational power (CPU) and memory (both RAM and disc); however, they can be easily deployed in many types of mobile and/or autonomous systems. An autonomous system depends only on locally available hardware and software; for example, it cannot use remote services available through the Internet. Despite the limitations of such hardware, novel multi-platform machine learning frameworks have made it possible to create implementations of face analysis algorithms that can be deployed across multiple types of hardware architectures and operation systems.

The aim of this paper is to design, train, evaluate, and implement a facial image processing algorithm which can perform face annotation based on images from a single RGB camera which has accuracy at the level of state-of-the art pattern recognition methods and which can be run on a low power consumption microcomputer. This goal is usually solved by training a classifier on another computer and then deploying the trained model onto a microcomputer which is supported by an additional co-processor. However, the inclusion of a co-processor increases the price of the hardware: depending on the platform we choose, the coprocessor may cost three times more than the microcomputer itself. This is a large increment of cost, especially if the system we want to create contains many separate microcomputers; for example, if each is a component of an individual patrolling robot. Due to this, the second assumption of this paper is that the proposed annotation method should be successfully deployed and run on a microcomputer without the aid of additional external computing modules. Of course, the classifier training procedure has to done on different hardware in order to be finished in reasonable time. In this paper, I will detail how this method has been developed, beginning from training on a large (over 200,000 images) data set, comparison with the state-of-the-art methods, and running it on a popular low power consumption device. I used popular hardware and machine learning platform solutions and all source code was published, such that the results I obtained can be reproduced. Developing and evaluating the proposed facial image annotation algorithm and its implementation, which is easily portable between various hardware and operating systems (virtually the same code works both on high-end PCs and microcomputers using the Windows and Linux platforms) and which is dedicated for low power consumption devices without coprocessors, is the main and novel contribution of this research.

## 2. Material and Methods

In this section, the data set on which the training and validation of the proposed face annotation algorithm has been done will be presented. Furthermore, I will discuss the proposed method and details of the state-of-the-art methods which the proposed method was compared to.

### 2.1. The Data Set

**Figure 1:**
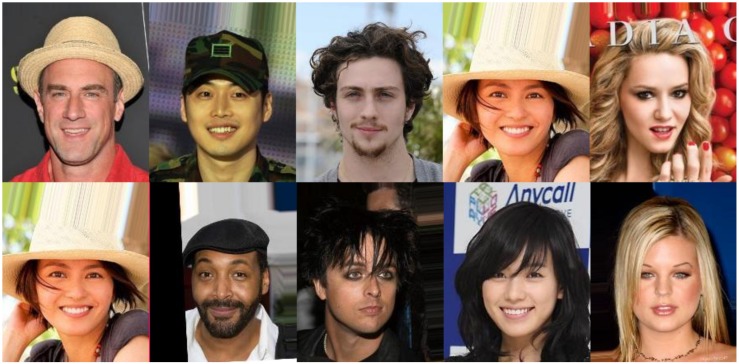
Several random pictures from the CelebA dataset, which was used in this research. The images were aligned and cropped such that eyes of each person are approximately in the same position. The faces were also rescaled, such that they have similar size.

The data set that was used in this research is Large-scale CelebFaces Attributes (CelebA) [19]. It can be downloaded from the Internet (http://mmlab.ie.cuhk.edu.hk/projects/CelebA.html). It contains over 200,000 pictures, mostly of actors, celebrities, sports players, and so on. Each image is described with 40 attribute annotations. As can be seen in Table 1, all attributes are binary, indicating the true/false condition of some annotations (classes); for example, gender, having a double chin, having a mustache, and so on. Although the distribution of images in the whole data set is random (there is no ordering of images that, for example, separates males from females), there is large imbalance [19] in the annotation classes: for example, only 4.15% of images have mustaches. This is an additional factor that makes classifier training more challenging. As data set pictures were taken “in the wild“, there is a large variety in terms of backgrounds and overall picture quality (contrast, saturation, and so on). In this research, I chose the “Align and cropped images” version of this data set, which was initially pre-processed by a procedure similar to the one described in Section 2.3. Figure 1 presents several samples from the data set.

This data set has been intensively used by many researchers to evaluate face annotation methods. Table 2 presents the accuracy (expressed in percentage) of some selected methods. As can be seen, the highest reported accuracy was 91.00% for the method of [22], which uses the MobileNetV2 DNN network. Therefore, I discuss this approach in next section and performed extensive comparisons of the results obtained by MobileNetV2 with those of the method proposed in this paper.

### 2.2. Mobilenetv2

Among the papers reviewed in the first section of this paper, the highest annotation accuracy on the CelebA data set has been reported by Luca et al. [22], where the authors used the DNN architecture MobileNetV2 [19], but without the top classification layers. The architecture of MobileNetV2 contains an initial convolutional layer with 32 filters, followed by 19 residual bottleneck layers. The kernel size is always 3 × 3 and dropout and batch normalization were used during training. The top layers were replaced by a dense (fully connected) network layer of size 1536 with the Rectified Linear Unit (ReLU) activation function:

(1) relu(x)=max(x,0)

That layer is followed by a batch normalization layer [29], followed by a dropout layer [30] and, finally, a dense layer of size 37 with sigmoid activation function:

(2) $$\sigma \left(x\right)=\frac{1}{1+e^{-x}}$$

The size of the input layer was a choice of the authors. The number of neurons in the output layer is equal to number of attributes that are classified by the network. The authors decided to skip three attributes of CelebA; namely Attractive, Pale_Skin, and Blurry. The output from the network is a single 37-dimensional vector, in which each value represents the classification of separate annotations of the input image. Facial images were scaled to a uniform resolution of 224 × 244 pixels in RGB colour space.

The network was trained on about 180,000 images from the CelebA data set. The rest of the data set was used for validation purposes. I adapted the same division of the data set for all methods evaluated in this research.

To potentially increase generalization ability, the data set was augmented by various transforms before training (i.e., rotation, width and height shifts, shearing, zooming, and horizontal flip), while the parameters of the transforms were randomly changed between samples. The training algorithm was AdaDelta [31], a gradient descent method that dynamically adapts over time using only first-order information. In AdaDelta, the update of parameter *y* in the *t*th iteration is $\Delta y_{t}$:

(3) $$y_{t+1}=y_{t}+\Delta y_{t}$$

(4) $$\left\{\begin{array}{c} \Delta y_{0}=0\\ \Delta y_{t}=-\frac{RMS{\left[\Delta y\right]}_{t-1}}{RMS{\left[g\right]}_{t}}g_{t} \end{array}\right.$$

where $RMS{\left[\Delta y\right]}_{t-1}$ is the root mean square of the previous updates of Δy up to time t−1 and $RMS{\left[g\right]}_{t}$ is root mean square of the previous square gradients *g* up to time *t*.

A loss function (optimization score function) was derived from the cosine similarity function defined as the dot (scalar) product between the normalized reference *r* and predicted *p* vectors:

(5) $$loss\left(r,p\right)=-\frac{r\cdot p}{\left|r\right|\left|p\right|}$$

where · is the dot product and $\left|r\right|$ is the norm of a vector *r*.

The metric to be evaluated by the model during training and testing was binary accuracy, which is defined as an averaged value of how many times the rounded values of binary predictions are equal to the actual value.

There were no obstacles to extending the output layer to 40 neurons and to train the network from scratch to classify all 40 attributes. I performed these extensions and training. The results are described in the Results and Discussion sections.

### 2.3. Histogram of Oriented Gradients Feature Descriptor and Neural Network

As was described in the previous section, an appropriately designed DNN can perform both face feature detection and annotation. It is, however, possible to perform those tasks using other approaches. Among the most popular methods for objects detection is the Histogram of Oriented Gradients (HOG) method [32]. This method is implemented by dividing the image window into small spatial regions (“cells”) and accumulating a local 1-D histogram of gradient directions (or edge orientations) over the pixels of each cell. In this research, I used HOG features combined with image pyramid and sliding window to handling various scales and object position and the SVM algorithm for classification. The face position estimator was created using dlib’s implementation of the work of Kazemi et al. [33] and it was trained on the iBUG 300-W face landmark data set [34]; the estimator is available online (https://github.com/davisking/dlib-models). In this particular implementation, a set of 68 landmarks on the face is detected, which can be used to align and rescale the face using basic image transforms such as translation, rotation, and scaling. All images in both the training and validation data sets were aligned and scaled to the same size (128 × 128 pixels) in RGB colour space. The images were used as an input to the classification algorithm, which was trained to perform image annotations. The process of classification was divided into 40 separate neural network classifiers. Each network was trained separately on each attribute using all images from the training data set, which were aligned by the previously mentioned procedure. Each of the 40 neural networks is a binary classifier, and they all shared the same architecture: a flattened input layer of size 128 × 128 × 3 that transforms the input RGB image into a vector, followed by a dense layer with 128 neurons which use the ReLU activation function and, finally, a dense layer with two neurons using softmax activation.

(6) $$softmax\left(x_{i}\right)=\frac{e^{x_{i}}}{\sum_{j}e^{x_{j}}}$$

The training algorithm was Adamax [35], the first-order gradient-based optimizer for stochastic objective functions that uses adaptive estimates of lower-order moments. The loss function was categorical cross-entropy, which returns a tensor calculated with a softmax function.

### 2.4. Eigenfaces with K-Nearest Neighbour Classifier

Another possible approach for facial image annotation is to perform facial image classification by using features derived from eigenfaces together with virtually any classification method. Similarly to Liton et al. [8], I selected the K-nearest neighbour classifier (KNN) as, contrary to NN approaches, it does not have generalization properties and it is possible to use the whole training data set as the reference dictionary. Therefore, this is a different approach to the considered problem than those discussed in this paper so far and the obtained results might be worth discussing.

In the first step, all images were aligned using the procedure described in Section 2.3, such that each facial images had a resolution of 128 × 128 pixels in RGB colour space. Then, of the 180,000 images in the training data set, 50,000 were used to calculate eigenfaces. Eigenfaces are p-dimensional vectors which are defined as eigenvectors of the covariance matrix of a facial image data set. Let us assume that each facial image has size n × m and that there are p images. In first step, we need to “flatten” each of these facial images by creating *p* column vectors $v_{i}$, in which the image columns are positioned one after another. These “flattened” vectors are columns of the matrix *D*, the dimension of which is [n∗m,p] (p, equal to number of images in data set, is the number of columns in the matrix). Next, a so-called mean face vector *f* is calculated, which is the average value of each row of the matrix *D*. Next, the mean face is subtracted from each column of *D* and the matrix D′ is created.

(7) $$D_{\left[n*m,p\right]}=\left[v_{1},\ldots ,v_{p}\right]$$

(8) $$D_{\left[n*m,p\right]}^{\prime }=\left[v_{1}-f,\ldots ,v_{p}-f\right]$$

The covariance matrix C is defined as:

(9) $$C_{\left[p,p\right]}=\frac{1}{p}\cdot {D^{\prime T}}_{\left[n*m,p\right]}\cdot D_{\left[n*m,p\right]}^{\prime }$$

Next, the eigenvalues and eigenvectors of C are found. To calculate the eigenfaces, D′ has to be multiplied by column matrix of eigenvectors. As the result, a column matrix of eigenvectors V of D′ is obtained. Those eigenvectors have the same eigenvalues as those calculated from C.

(10) $$V_{n*m,p}=D_{\left[n*m,p\right]}^{\prime }\cdot V_{C[p,p]}$$

where $V_{C[p,p]}$ are eigenvectors of *C*.

Then, the eigenvectors are sorted according to descending values of their corresponding eigenvalues (all eigenvalues of the covariance matrix are real and positive). A facial image can be encoded as a linear combination of eigenvectors. To find those coefficients, the transpose of $V_{n*m,p}$ has to be multiplied by the input flattened facial image $v_{j}$ subtracted by the mean face.

(11) $$e_{j}={V^{T}}_{\left[n*m,p\right]}\cdot \left(v_{j}-f\right)$$

The inverse procedure, which recalculates encoding to the facial image, is:

(12) $$v_{j}^{\prime }=\left(V_{\left[n*m,p\right]}\cdot e_{j}\right)+f$$

**Figure 2:**
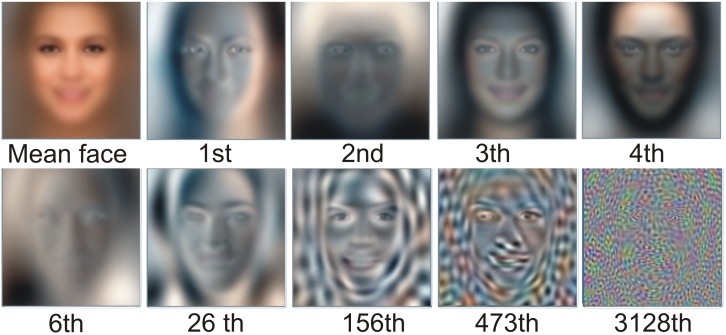
A mean face (in which pixels are averaged values of whole training data set of 50,000 images), then first four eigenfaces, sixth eigenface (six first eigenfaces explains 50% of variance), 26th (26 first eigenfaces explains 75% of variance), 156th (156 first eigenfaces explains 90% of variance), 473rd (473 first eigenfaces explains 95% of variance), and 3128th (3128 first eigenfaces explains 99% of variance).

**Figure 3:**
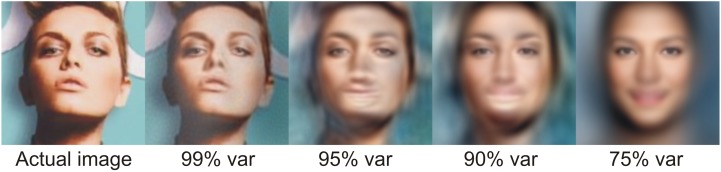
Actual (input) image and its reconstruction using various numbers of eigenfaces, which describe 99%, 95%, 90%, and 75% of variance, respectively.

The percentage of variance explained by the r first eigenvectors can be calculated as the cumulative sum of the r first eigenvalues. Eigenfaces with lower indices (those which explain more variance) have a similar role to the low-frequency coefficients in frequency-based decomposition. Those with higher indices are responsible for high frequencies (i.e., details). These facts can be easily spotted in Figure 2, which presents a mean face (in which pixels are averaged values of whole training data set of 50,000 images), then first four eigenfaces, sixth eigenface (six first eigenfaces explains 50% of variance), 26th (26 first eigenfaces explains 75% of variance), 156th (156 first eigenfaces explains 90% of variance), 473rd (473 first eigenfaces explains 95% of variance), and 3128th (3128 first eigenfaces explains 99% of variance). As can be seen, the last eigenfaces have more detail, while the first few present various global properties of the image, such as lighting, face shapes, and so on. Before classification, the number of eigenfaces required has to be determined, in order for the decoded image to preserve the face attributes that we are interested in. The less eigenfaces required to keep that information, the less dimensional the classification problem becomes. As can be seen in Figure 3, the individual facial features of this particular person start to become visible when at least 95% of variance is present (at least 473 coefficients), while the face is recognizable when at least 99% of variance is present (at least 3128 coefficients). Based on the above discussion, before using K-NN classifier, both the training and validation data sets were recalculated to 3128-dimensional space, according to Equation (11). In K-NN, the whole training set of 180 000 faces was used as reference images for classification.

## 3. Results

**Figure 4:**
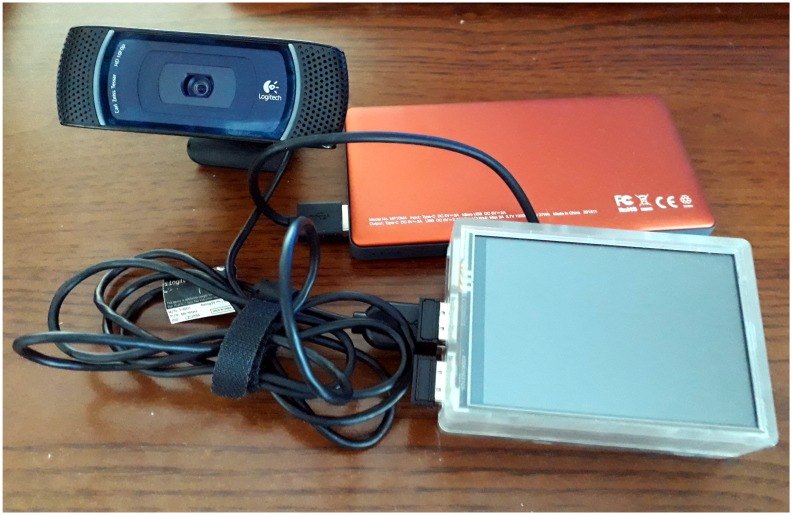
The assembled microcomputer that was used in this research. It consists of a Raspberry Pi 3 model B+ in a semi-transparent case with a 3.5″ LCD touchscreen, a Logitech Webcam C920 HD Pro, and a powerbank (5 V, 3 A).

I implemented all solutions from the previous section in Python 3.6. The method from Section 2.2 was implemented by the authors; I only had to make some small adjustments. Among the most important packages used were: Tensorflow 2.1, for machine learning with GPU support in order to speed up network training; the DNN Keras 2.3.1 library; the dlib 19.8.1 package for face detection and aligning; the sklearn package for KNN; and OpenCV-python 4.2.0.32 for general-purpose image processing. For algorithm training and evaluation, I used a PC with an Intel i7-9700F 3.00 Ghz CP, 64 GB RAM, an Intel i7-9700F 3.00 Ghz CPU, and a NVIDIA GeForce RTX 2060 GPU on Windows 10 OS. The trained network was then deployed on a Raspberry Pi 3 model B+ microcomputer (1.4 GHz, 1 GB RAM on Raspbain 4.19.75-v7). The Raspberry Pi had Python 3.6, Tensorflow 2.0, Keras 2.3.1, dlib 19.19.99, and OpenCV-python 4.1.1 installed. The library versions I used on the PC and microcomputer differed, as the platforms support different distributions of packages and the installation procedure is different (on PC, most packages were installed with PIP while on Raspberry, I had to compile them from source). Furthermore, GPU support for Tensorflow 2.1 requires certain distributions of packages. The process of installation and configuration of programming and runtime environment is, however, out of the scope of this paper. After successful installation of Python runtime, the neural networks that were trained on PC were loaded onto the Raspberry microcomputer without problems. The video sensor I used was a Logitech Webcam C920 HD Pro plugged into the USB 2.0 interface of the Raspberry Pi. The camera was used in test whether the microcontroller is capable of performing annotations of incoming video frames. The microcomputer was also equipped with a 3.5″ LCD touchscreen and powered with a powerbank (5 V, 3 A). The price of the microcomputer was about 40$. Figure 4 presents a picture of the assembled microcomputer which was used in this research.

As was described in Section 2.2, the CelebA data set was divided into a training data set containing 180,000 ( 88% of CelebA) images and a validation data set that contained the rest of the data set ( 12% of CelebA). As the validation data set contained over 22,000 images, leave-one-out cross validation was not necessary. The same division was also used in [36].

In the case of the DNN proposed by Sandler et al. [36], I used two versions of it. The first version was exactly the DNN version provided by the authors (https://github.com/Luca96/face-clustering), which is named DNN 37 in Table 3 and Table 4. This network annotates 37 attributes: the Attributes ’Attractive’, ’Pale_Skin’, and ’Blurry’ were skipped. The second version of this network had all 40 features and was trained from scratch for the purposes of this paper, which is named DNN 40 in Table 3 and Table 4. I set the batch size to 32 and used 12 epochs. The whole training procedure, as described in detail in Section 2.2, took less than 5 h.

The HOG–NN solution described in Section 2.3 used the same training and validation data set. It is named NN 40 in Table 3 and Table 4. Training of each of 40 networks took about 45 min.

Eigenfaces for the KNN classifier were generated from 50,000 images from the training dataset. I took eigenvectors that explained approximately 99% of the variance (3128 eigenfeatures); this choice is explained in Section 2.4. I tested 1-NN and 3-NN classifiers, which are named 1-NN and 3-NN in Table 3 and Table 4. Their reference data was the full training data set consisting of 180,000 images. The validation dataset was the same as that used for the previous classifiers.

All source code that was written for this paper is available online (https://github.com/browarsoftware/FaceImagesAnnotation).

## 4. Discussion

As can be seen in Table 3, the proposed method, which used the HOG face detector and the set of 40 NNs which performed annotation of each face attribute separately had the highest accuracy of all of the validated classifiers. These results may, however, be misleading before examining the results in Table 4. As can be seen in Table 4, no classifier has the highest TPR and TNR in all examined attributes. The 3-NN and 1-NN classifiers had worse results than NN 40 most of the time; however, there were some attributes that were classified better. For example, Wearing_Necklace and Wavy_Hair were not classified to separate classes by the NNs at all. These situations happen to be a limitation of the “classical” (not deep) NN models. The averaged TPR and TNR strongly indicate that the classes were imbalanced, resulting in a large difference in averaged TPR and TNR value for each attribute of the data set. It is not generally true that TPR always had a smaller value than TNR (see, for example, the Young attribute); however, it caused a disproportion in all classifiers, due to which TPR was over two times smaller than TNR. The reason why TNR typically had a higher value than TPR in unbalanced classes is that, in the training data set, there were much more negative than positive examples of particular attributes. We can easily conclude that none of these methods, even the state-of-the-art [22] were capable of classifying the data set with appropriate balance; that is, in a way that both TNR and TPR for each class had similarly high values (i.e., at the level of averaged accuracy of the certain classifier). The highest averaged TPR was obtained for NN 40 (47.54), the second highest was DNN 40 (46.87); the difference between those two was 0.67. The third highest TNR was obtained for NN 37 (43.75); the difference between this one and the second was 3.12. In case of TNR, the highest value was obtained for 30 DNN (95.98), the second highest was DNN 40 (94.74); the difference between those two was 1.24. The third highest TNR was obtained for NN 40 (92.12); the difference between this and the second was 2.62. Taking both averaged TPR and TNR into account, we can conclude that DNN 40 might be slightly more effective than NN 40.

The real difference between these two models became visible while deploying them on a microcomputer with very limited RAM. In the case of a microcontroller with 1 GB RAM (although mobileNetV2 has been specifically tailored for mobile and resource constrained environments) both DNN 37 and DNN 40 failed at loading into the tested low power consumption device. On the other hand, it was possible to successfully run NN 40 on the evaluated device, gradually loading and unloading each of the networks after it performed annotation of a single attribute. It is also possible to perform online annotation of human faces captured by the USB camera sensor connected to the device, as described in Section 3. The source code for this application, together with the weights of the 40 NNs can be downloaded from the same GitHub repository as the rest of the source code prepared for this paper.

The resource usage of the NN 40 method on PC and low power consumption microcomputer that were presented in Section 3 was evaluated. I used the approach in which each NN is separately loaded into memory, performs labelling, and then is unloaded from memory. It is done in order to not to exceed the 1 GB memory of the microcomputer. The time required for HOG face detection (in resolution 640 × 480), facial image aligning, loading single network model, and predicting face features were measured, as well as the total time for processing one acquired frame (which includes all of the previous operations) and labelling all 40 features. I also measured the RAM memory usage and CPU usage of the Python process. The results averaged from 20 facial images acquired by USB camera are presented in Table 5.

PC HOG face detection was among most time-consuming operation in the image processing pipeline. Face aligning, however, was very fast (4 milliseconds). The total time for face detection, aligning, and labelling of all 40 features by loading and unloading the NN models individually was about 4.8 s. The process occupied 1.1 GB of RAM and used 20.5% of CPU power. Annotation (predicting) of a single feature took 27 milliseconds. When evaluating microcomputer processing, the times were significantly longer. This situation can be easily explained when we compare memory usage: it was only 0.3 GB. This is because the operating system performs memory swapping in order to not to exceed 1 GB of RAM, which is a limit for the whole system. The swapping was performed on an SD memory card, so it was relatively fast; however, RAM is important limitation in microcontrollers. The CPU usage was about 100%. This, however, did not freeze the microcomputer; one can easily use other applications at the same time while not expiring lags as the examined CPU has a multicore architecture. Annotation (prediction) time was nearly 10 times slower than on PC (0.2 s per feature). Face aligning was nearly 15 times slower than on PC. The annotation of all 40 features, individually loading and unloading models, took about 2 min. This was quite a long time, compared to PC; however, we have to remember that the efficiency of the classifier on the microcomputer is the same as in PC and the price of PC used in this research was about 50 times higher (!) than the microcomputer. Furthermore, one can easily linearly scale the performance of the NN 40 solution by excluding classifiers that might be unnecessary for the certain task. Both on PC and microcontroller, the proposed solution does not operate in real time (it was not higher than video frame acquisition time).

## 5. Conclusions

In this paper, I proposed an NN classifier designed for human face attribute annotation, which is capable of running on relatively cheap, low power consumption systems. The proposed solution, consisting of a HOG face detector and a set of neural networks, has comparable averaged accuracy and averaged TPR and TNR to state-of-the-art DNNs; however, in contrast to the tested DNNs, it is possible to deploy the proposed solution for limited RAM memory applications, such as microcomputers without additional co-processors. Additional tests were carried out, proving that the implementation of the proposed method can perform annotation of facial images acquired by an RGB camera, reusing virtually the same source code and neural network weights, both on a high-end PC and a microcomputer. The results obtained can be easily reproduced, as both the data set and source code are available online. The presented results are especially important for researchers and engineers who develop autonomous systems based on low power consumption, general-purpose microcomputers which need to perform autonomous classification tasks without fast internet communication (i.e., without outsourcing image recognition to remote servers).

## Figures and Tables

**Table 1:** The distribution of binary annotations in the data set. For example, 4.15% of images contain people with mustaches.

Feature Name	True	False
X5_o_Clock_Shadow	11.11%	88.89%
Arched_Eyebrows	26.7%	73.3%
Attractive	51.25%	48.75%
Bags_Under_Eyes	20.46%	79.54%
Bald	2.24%	97.76%
Bangs	15.16%	84.84%
Big_Lips	24.08%	75.92%
Big_Nose	23.45%	76.55%
Black_Hair	23.93%	76.07%
Blond_Hair	14.8%	85.2%
Blurry	5.09%	94.91%
Brown_Hair	20.52%	79.48%
Bushy_Eyebrows	14.22%	85.78%
Chubby	5.76%	94.24%
Double_Chin	4.67%	95.33%
Eyeglasses	6.51%	93.49%
Goatee	6.28%	93.72%
Gray_Hair	4.19%	95.81%
Heavy_Makeup	38.69%	61.31%
High_Cheekbones	45.5%	54.5%
Male	41.68%	58.32%
Mouth_Slightly_Open	48.34%	51.66%
Mustache	4.15%	95.85%
Narrow_Eyes	11.51%	88.49%
No_Beard	83.49%	16.51%
Oval_Face	28.41%	71.59%
Pale_Skin	4.29%	95.71%
Pointy_Nose	27.74%	72.26%
Receding_Hairline	7.98%	92.02%
Rosy_Cheeks	6.57%	93.43%
Sideburns	5.65%	94.35%
Smiling	48.21%	51.79%
Straight_Hair	20.84%	79.16%
Wavy_Hair	31.96%	68.04%
Wearing_Earrings	18.89%	81.11%
Wearing_Hat	4.85%	95.15%
Wearing_Lipstick	47.24%	52.76%
Wearing_Necklace	12.3%	87.7%
Wearing_Necktie	7.27%	92.73%
Young	77.36%	22.64%

**Table 2:** The accuracy (expressed in percentage) of various state-of-the-art methods in the task of image annotation on the CelebA data set.

Method	Accuracy
LNet [19]	85.00
LNet+ANet [1]	87.00
CNN+SVM [20]	89.80
ft +Color+LBP+SIFT [21]	90.22
MobileNetV2 [22]	91.00

**Table 3:** Accuracy (expressed in percentage) of each classifier on each attribute.

Feature	DNN 37	DNN 40	NN 40	3-NN	1-NN
X5_o_Clock_Shadow	93.32	93.34	90.78	88.05	85.5
Arched_Eyebrows	78.38	81.08	77.84	69.64	67.16
Attractive	NA	78.46	75.43	66.77	64.15
Bags_Under_Eyes	80.81	83.44	82.32	77.6	74.21
Bald	98.84	98.39	97.76	97.66	96.86
Bangs	94.64	95	93.1	89.19	86.83
Big_Lips	69.31	70.3	69.29	65.46	62.72
Big_Nose	80.45	82.1	81.92	77.28	73.75
Black_Hair	76.63	72.35	82.15	71.77	68.45
Blond_Hair	87.85	88.13	93.26	89.02	87.28
Blurry	NA	95.48	95.29	92.04	86.49
Brown_Hair	82	81.86	84.59	76.81	71.87
Bushy_Eyebrows	87.6	89.53	90.41	84.38	80.14
Chubby	95.25	95.15	94.84	94.35	92.95
Double_Chin	96.11	95.64	95.85	95.07	93.43
Eyeglasses	99.52	99.32	97.87	94.4	93.76
Goatee	96.97	96.69	96.1	95.12	93.3
Gray_Hair	97.62	96.44	97.32	96.65	95.41
Heavy_Makeup	67.8	80.38	85.53	75.64	72.48
High_Cheekbones	79.51	79.4	82.68	65.19	62.15
Male	92.39	95.22	94.58	83.11	80.71
Mouth_Slightly_Open	92.03	91.67	83.3	62.03	60.65
Mustache	96.59	96.65	96.5	96.05	94.6
Narrow_Eyes	86.88	86.09	86.66	83.75	80.02
No_Beard	95.06	94.09	92.31	85.9	83.55
Oval_Face	70.81	71.89	71.87	64.49	61.98
Pale_Skin	NA	31.95	96.55	95.51	94.76
Pointy_Nose	74.75	71.88	73.81	67	63.6
Receding_Hairline	92.83	92.12	91.95	90.31	87.41
Rosy_Cheeks	92.83	92.83	93.7	91.62	88.95
Sideburns	97.41	97.34	96.37	95.31	93.28
Smiling	90.97	88.33	88.16	66.01	63.2
Straight_Hair	81.57	80.16	78.8	72.99	68.23
Wavy_Hair	77.28	78.75	73.29	67.34	64.88
Wearing_Earrings	84.58	87.71	81.45	77.15	73.68
Wearing_Hat	98.74	98.24	97.97	96.71	96.36
Wearing_Lipstick	67.86	90.27	90.84	79.21	75.23
Wearing_Necklace	86.31	86.28	85.92	81.89	78.24
Wearing_Necktie	96.7	95.44	94.75	92.95	91.05
Young	86.34	84.17	83.29	76.38	73.52
Averaged accuracy	87.15	86.59	88.20	82.19	79.57

**Table 4:** True positive rates (TPR) and true negative rates (TNR) of each classifier on each attribute (expressed in percentage).

	DNN 37		DNN 40		NN 40		3-NN		1-NN	
**Feature**	**TPR**	**TNR**	**TPR**	**TNR**	**TPR**	**TNR**	**TPR**	**TNR**	**TPR**	**TNR**
X5_o_Clock_Shadow	38.97	99.35	45.54	98.65	58.81	94.39	22.77	95.43	31.76	91.58
Arched_Eyebrows	30.57	97.39	53.52	92.04	63.8	83.61	39.48	82.04	43.02	77.08
Attractive	NA	NA	66.05	90.66	89.59	60.82	68.51	64.96	65.85	62.4
Bags_Under_Eyes	71.52	83.18	31.67	96.59	39.98	93.25	18.98	92.74	27.03	86.4
Bald	74.23	99.38	40.9	99.63	17.66	99.45	6.59	99.59	17.07	98.55
Bangs	69.73	99.24	74.27	98.83	66.94	97.87	48.73	96.57	50.54	93.45
Big_Lips	8.98	98.63	31.54	89.14	20.57	92.98	19.94	87.59	27.86	79.67
Big_Nose	64.3	84.79	53.28	89.85	34.53	94.74	22.59	92.07	29.36	85.76
Black_Hair	23.88	96.29	10.88	95.27	49.93	94.06	35.61	85.13	40.15	78.91
Blond_Hair	9.14	99.95	11.88	99.85	62.06	98.22	47.56	95.61	50.72	93.09
Blurry	NA	NA	58.12	97.47	0.4	99.95	10.49	96.04	18.46	89.83
Brown_Hair	0	99.96	0.03	99.78	49.78	92.32	29.25	87.37	36.85	79.65
Bushy_Eyebrows	4.41	99.98	24.9	99.15	44.12	97.43	15.62	94.81	25.25	88.47
Chubby	14.18	99.79	18.62	99.43	25.56	98.66	9.16	99.04	16.65	97.16
Double_Chin	19.93	99.76	4.71	99.99	18.12	99.51	8.58	99.14	17.29	97.01
Eyeglasses	94.49	99.87	92.94	99.76	76.45	99.27	11.42	99.78	19.06	98.61
Goatee	45.9	99.42	71.91	97.88	33.29	99.06	10.36	99.11	17.27	96.88
Gray_Hair	57.08	98.95	68.87	97.35	38.32	99.22	21.36	99.07	30.34	97.51
Heavy_Makeup	20.61	99.92	53.54	98.64	92.95	80.28	67.74	81.24	64.38	78.22
High_Cheekbones	59.58	98.04	59.25	98.12	78.46	86.77	61.12	69.15	58.98	65.22
Male	99.21	88.1	96.09	94.68	90.54	97.04	72.84	89.38	71.71	86.21
Mouth_Slightly_Open	90.22	93.81	91.17	92.16	89.5	77.12	55.35	68.67	55.18	66.09
Mustache	19.69	99.68	27.2	99.44	22.6	99.32	3.04	99.61	10.12	97.83
Narrow_Eyes	18.83	98.76	9.84	99.41	21.93	97.74	5.18	97.2	13.47	91.42
No_Beard	98.7	73.78	95.33	86.85	97.04	64.52	95.77	27.92	91.7	35.7
Oval_Face	2	99.69	6.96	99.13	21.65	93.58	31.01	78.97	38.07	72.32
Pale_Skin	NA	NA	97.14	29.08	45.85	98.84	2	99.75	11.16	98.55
Pointy_Nose	21.32	96.13	2.09	99.8	35.01	89.63	32.96	80.88	38.97	73.64
Receding_Hairline	41.38	97.6	14.46	99.32	8.2	99.64	12.05	97.5	21.66	93.44
Rosy_Cheeks	0	100	0	100	24.64	99.13	13.3	97.78	22.25	94.2
Sideburns	52.92	99.57	67.71	98.78	37.97	99.21	10.7	99.43	17.91	96.96
Smiling	84.2	97.74	78.33	98.35	89.76	86.49	64.87	67.2	62.34	64.1
Straight_Hair	29.02	95.53	24.75	94.88	0	100	20.28	87.16	29.72	78.59
Wavy_Hair	40.46	98.36	48.3	96.19	50.34	86.66	44.61	80.57	46.31	75.69
Wearing_Earrings	27.73	99.38	52.34	96.92	24.93	96.57	20.81	92.22	29.32	85.54
Wearing_Hat	79.5	99.58	76.16	99.21	54.05	99.65	14.53	99.85	19.93	99.28
Wearing_Lipstick	38.49	99.92	84.81	96.23	88.66	93.34	78.7	79.79	73.8	76.88
Wearing_Necklace	1.05	99.95	1.6	99.83	0	100	15	92.86	23.48	87.21
Wearing_Necktie	72.27	98.55	39.81	99.63	42.67	98.66	19.2	98.49	28.44	95.76
Young	94.34	61.41	88.21	71.58	95.01	45.89	91.01	29.72	85.33	35.84
Averaged	43.75	95.98	46.87	94.74	47.54	92.12	31.98	87.04	36.97	83.52

**Table 5:** Results of resource usage of the NN 40 method averaged from 20 measurements. Columns represents (from left from right): time of HOG face detection, facial image aligning, loading single network model, predicting face feature, total time for processing one acquired frame (including all previous operations) and labelling of all 40 features, RAM memory usage of Python process, and CPU usage of Python process.

Computer	HOG [s]	Aligning [s]	Loading [s]	Predicting [s]	Total [s]	RAM [GB]	CPU [%]
PC	0.429	0.004	0.053	0.027	4.816	1.139	20.5
Raspberry	10.485	0.059	1.668	0.225	107.5	0.311	100
